# Evaluation of a treatment planning system developed for clinical boron neutron capture therapy and validation against an independent Monte Carlo dose calculation system

**DOI:** 10.1186/s13014-021-01968-2

**Published:** 2021-12-24

**Authors:** Naonori Hu, Hiroki Tanaka, Ryo Kakino, Syuushi Yoshikawa, Mamoru Miyao, Kazuhiko Akita, Kayako Isohashi, Teruhito Aihara, Keiji Nihei, Koji Ono

**Affiliations:** 1 Kansai BNCT Medical Center, Osaka Medical and Pharmaceutical University, Osaka-fu Takatsuki-shi Daigakumachi 2-7, Takatsuki, Japan; 2 Institute for Integrated Radiation and Nuclear Science, Kyoto University, Kyoto, Japan; 3 Central Department of Radiology, Osaka Medical and Pharmaceutical University Hospital, Takatsuki, Japan; 4 Department of Radiation Oncology, Osaka Medical and Pharmaceutical University Hospital, Takatsuki, Japan

**Keywords:** Boron neutron capture therapy, Treatment planning system, Commissioning, Monte Carlo simulation

## Abstract

Boron neutron capture therapy (BNCT) for the treatment of unresectable, locally advanced, and recurrent carcinoma of the head and neck cancer has been approved by the Japanese government for reimbursement under the national health insurance as of June 2020. A new treatment planning system for clinical BNCT has been developed by Sumitomo Heavy Industries, Ltd. (Sumitomo), NeuCure® Dose Engine_._ To safely implement this system for clinical use, the simulated neutron flux and gamma ray dose rate inside a water phantom was compared against experimental measurements. Furthermore, to validate and verify the new planning system, the dose distribution inside an anthropomorphic head phantom was compared against a BNCT treatment planning system SERA and an in-house developed Monte Carlo dose calculation program. The simulated results closely matched the experimental results, within 5% for the thermal neutron flux and 10% for the gamma ray dose rate. The dose distribution inside the head phantom closely matched with SERA and the in-house developed dose calculation program, within 3% for the tumour and a difference of 0.3 Gy_w_ for the brain.

## Background

The world’s first accelerator based epithermal neutron source for clinical boron neutron capture therapy (BNCT) was designed and developed by Sumitomo Heavy Industries, in collaboration with Kyoto University BNCT research group [1, 2]. The system was installed in December 2008 and neutron production tests began in March 2009. By December 2010, beam characterisation and in-vitro/in-vivo tests were performed. In 2012, a phase I and II clinical trials for recurrent malignant gliomas were performed [3], followed by clinical trials for head and neck cancer in 2016.

In 2016, the same type of accelerator was installed at the Osaka Medical and Pharmaceutical University, Kansai BNCT Medical Center. The system obtained approval of a new medical device for manufacturing and sales of an accelerator BNCT system (NeuCure® System) and the dose calculation program (NeuCure® Dose Engine, henceforth NeuCure) from the Japanese Ministry of Health, Labor and Welfare on 11th March 2020. These products were approved for reimbursement for unresectable, locally advanced, and recurrent carcinoma of the head and neck region covered by the Japanese national health insurance system on 1^st^ June 2020.

Recently, Kumada et al., published a review article summarising the BNCT treatment planning system (TPS) workflow and provided a list of the major TPS used for BNCT [4]. A commonly used TPS for BNCT are SERA (Simulation Environment for Radiotherapy Applications) [5], which was developed by INL/Montana State University and NCTPLAN [6], which was developed by Harvard-MIT and the CNEA group. The performance comparison between these two TPS were performed by Wojnecki and Green [7]. Goorley et al. performed a phantom study using a general-purpose Monte Carlo radiation transport code MCNP to evaluate the effects of material composition, kinetic energy released in matter (KERMA) factors, model mesh size and beam energy on dose profiles. The result indicated for large voxel sizes, deviations in KERMA rates were unacceptability large [8]. SERA, due to the limitation in the memory usage, has a large voxel size (10 mm). Other issues include complicated entry of region of interest and patient tissue information, and poor input–output usability (SERA cannot process data in DICOM file format).

Due to the above limitations of the currently available TPS for BNCT, a new TPS for the purpose of insurance covered BNCT was required (since SERA and NCT are not approved as a medical device by any authorities). The NeuCure, developed by Sumitomo, became the world’s first BNCT dose calculation program approved as a medical device for the purpose of clinical BNCT. The system is installed in a general-purpose personal computer and used in combination with the radiotherapy planning program RayStation (product of RaySearch Laboratories) installed in the same PC. Patient contour information and irradiation conditions are set on RayStation and the information are recorded in DICOM format. This information is used as input data in NeuCure and the dose distribution and monitor units are calculated. The Particle and Heavy Ion Transport code System (PHITS) is used to simulate both the neutron and photon transport [9]. PHITS is a general-purpose Monte Carlo particle transport simulation code system written in Fortran. The calculated data are sent back to RayStation and the dose distribution and dose volume histogram are displayed graphically.

To verify and commission the NeuCure system, experimental measurement of the neutron and gamma ray distribution inside a phantom is necessary. Furthermore, a comparison with an independent calculation is recommended, particularly as the dose calculation component and the patient voxelisation component is developed by separate companies and the integration of the two software needs to be thoroughly examined. The purpose of this study is to validate the neutron and gamma ray distribution simulated by NeuCure with experimental measurements and to perform a comparison against an independent calculation system. Furthermore, a comparison with SERA was performed. Despite the above limitations of SERA, the clinical trials which were carried out between 1990 to 2014 at the Institute for Integrated Radiation and Nuclear Science (KURNS) using the reactor based BNCT system [10–13], and the abovementioned phase I and II clinical trials for brain [14] and head and neck cancer using the accelerator-based BNCT system all utilised SERA for the dose calculation. Many of the groundwork has been laid out by SERA, so comparing the new TPS with SERA will add confidence to the calculation model.

## Materials and methods

### Dose calculation systems

1. NeuCure
  The current version of NeuCure utilises PHITS version 3.2 for particle transport. The BNCT dose was calculated using the simulated neutron and gamma ray fluxes, along with the corresponding KERMA and dose conversion factors [15]. The Japanese Evaluated Nuclear Data Library 4.0 (JENDL 4.0) developed by Japan Atomic Energy Agency (JAEA) was used [16]. The system runs on Windows 10 and the Monte Carlo simulation was performed using a central processing unit (CPU) parallel computing (Intel Xeon Gold 6246R processor 3.4 GHz, 32 GB RAM). RayStation version 9A was used to convert the CT images into a voxel model with both the mass density and the material information stored in each voxel.
2. SERA
  SERA version 1CO was used for this study. SERA uses a reconstruction technique based on a pixel-by-pixel uniform volume element called “univel”. The radiation transport is based on multigroup neutron and photon cross sections that were processed from the ENDF/B V and ENDF/B VI [7]. Further details of the SERA program can be found elsewhere [17, 18].
3. Independent PHITS model

**Fig. 1:**
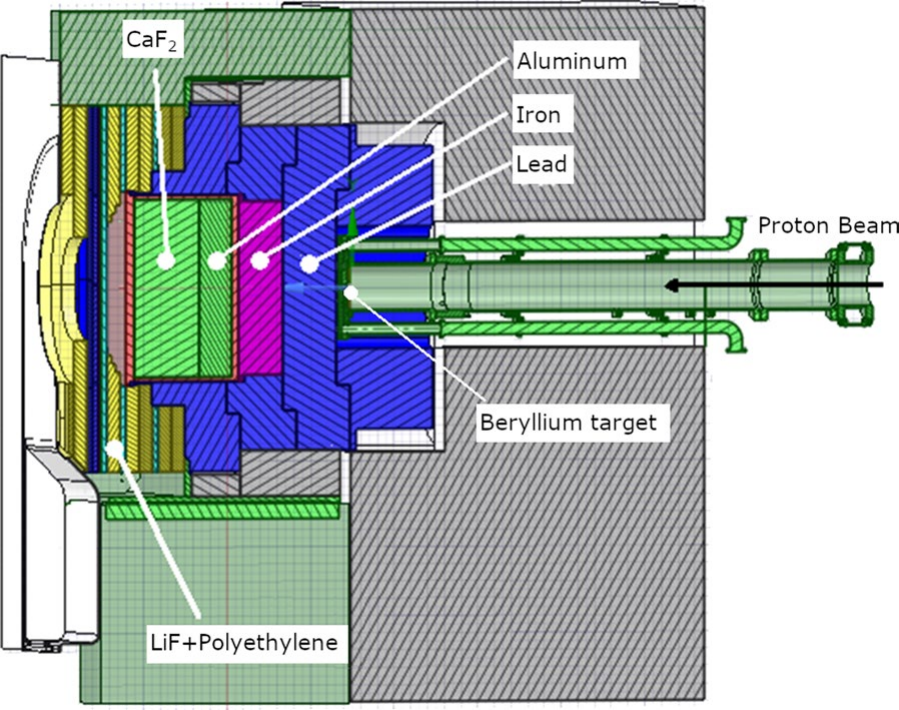
Cross-sectional diagram of the BSA of the NeuCure BNCT accelerator

The neutrons and photons generated when a 30 MeV proton beam striking a beryllium target was simulated. The beam shaping assembly (BSA) shown in Fig. 1 was modelled according to the design and the neutron and gamma ray spectrum at the lead surface (red dotted line) was tallied. A total of 51 bins for the beam angle (between 0 and 90 degrees) with the corresponding particle generation probability were tallied. After the neutron and gamma ray spectrum were obtained, a planar source (originating from the lead surface) was generated and used for subsequent simulations to speed up the Monte Carlo simulation. The collimator material (yellow region) was set as polyethylene mixed with natural LiF. Further details of the BSA and the neutron spectrum can be found elsewhere (Tanaka et al.2009). The geometry modelling and particle transport were performed using PHITS version 3.24. The same nuclear data library (JENDL4.0) and KERMA coefficients to NeuCure were used to calculate the dose. The neutron energy range was set to:

- Thermal neutron range: 10^–20^ MeV < *E*_*n*_ ≤ 5.3 × 10^–7^ MeV
- Epithermal neutron range: 5.3 × 10^–7^ MeV < *E*_*n*_ ≤ 4 × 10^–2^ MeV
- Fast neutron range: 4 × 10^–2^ MeV < *E*_*n*_ ≤ 30 MeV

An upper limit of 40 keV for epithermal neutrons was selected based the study performed by Blue et al., which showed the relative biological effectiveness (RBE) at this energy being approximately the same as the thermal neutron energy region [19]. Also, Yanch et. al defined a useful energy range (neutron energies capable of effectively treating to a depth of 7 cm in brain tissue) to be 4 eV to 40 keV [20].

### Experimental measurement of the thermal neutron flux, fast neutron component and gamma ray dose rate

**Fig. 2:**
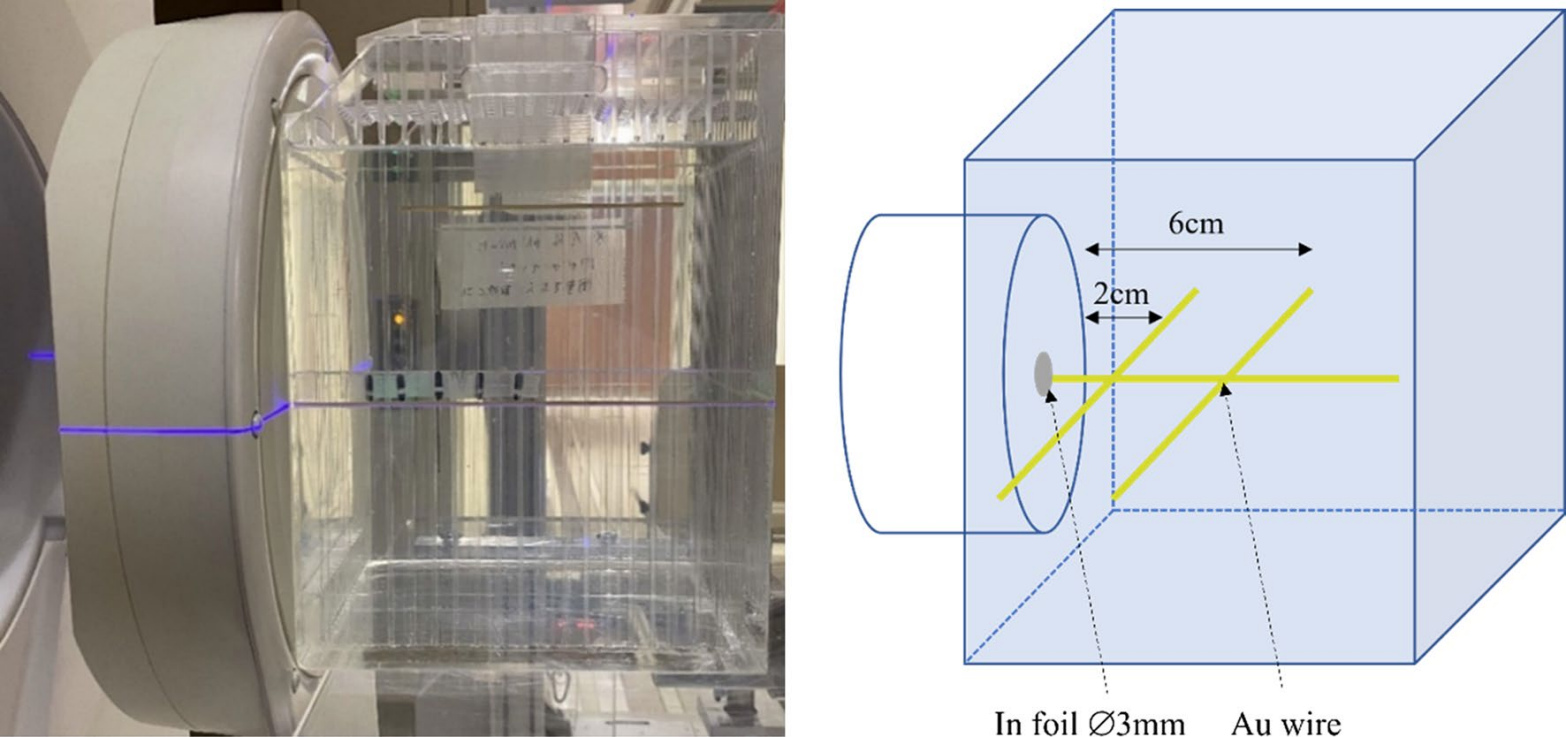
Left: QA phantom filled with distilled water used for routine QA with TLDs placed along the central beam axis for gamma ray dose rate measurement. Right: Schematic of the experimental set up illustrating the gold wires placed parallel and perpendicular (at 2 cm and 6 cm depth) to the beam axis to measure the thermal neutron flux and the indium foil placed at the centre of the field in front of the water phantom

A common detector for characterising neutron spectrum is activation foils. Gold is generally used for measuring both the thermal and epithermal neutron flux, as recommended by the IAEA TecDoc 1223 [21]. To distinguish between the thermal and epithermal neutron fluxes, measurements were performed both with bare and cadmium covered gold. The QA phantom filled with distilled water was used for this study (Fig. 2). For the measurement of the central axis thermal neutron flux, a thin gold wire (diameter of 0.25 mm with a length of 10 cm with a 99.95% purity, The Nilaco Corporation, Tokyo, Japan) was placed in the water phantom along the central beam axis with and without a cadmium tube cover, manufactured by Shieldwerx™ (approximately 1.3 mm and 2.3 mm for the inner and outer diameter, respectively and a length of 10 cm). For the off-axis profile measurement, same method was applied however the gold wire was placed perpendicular to the beam axis. A total proton charge of 0.3 C and 0.6 C were delivered for the gold wire only and gold wire covered with cadmium cover, respectively. After neutron irradiation, the activated gold wire was cut into small lengths (5–10 mm) and the gamma rays emitted from the activated gold foil was measured using a high purity germanium detector (ORTEC ICS-P4). The reaction rate per unit charge of the gold sample was calculated using the expression below.

$$R=\frac{\lambda C}{\epsilon \gamma {e}^{-\lambda {T}_{C}}\left(1-{e}^{-\lambda {T}_{m}}\right){\sum }_{i=1}^{n}\left(\frac{{Q}_{i}}{\Delta t}\left(1-{e}^{-\lambda \Delta t}\right){e}^{-\lambda \left(n-i\right)\Delta t}\right)}$$

where $\epsilon$ is the detection efficiency of the detector of the gamma rays emitted from ^198^Au, ɣ is the gamma ray emission rate from ^198^Au decay, λ is the decay constant of ^198^Au, T_c_ is the time from the irradiation to the start of the measurement, T_m_ is the measurement time, C is the peak count due to the detector measured gamma rays emitted from ^198^Au and Q_i_ is the electric charge irradiated on the target at each interval, Δt. For the measurement of fast neutrons, indium foil was used (diameter of 3 mm with a thickness of 0.1 mm with a 99.99% purity, The Nilaco Corporation). ^115^In (natural abundance of 95.7%) is excited by the (n,n’) process producing ^115m^In, which returns to the ground state by emitting a 340 keV gamma ray. Indium also reacts to low energy neutrons that produce both beta and gamma rays with different energies, making the measurement of the 340 keV difficult. To minimise these reactions, the indium foil was covered with cadmium to shield the low energy neutrons. The cadmium covered indium foil was placed at the centre of the water phantom surface (where the fast neutron flux is the highest). A total proton charge of 1.0 C was delivered. After irradiation, the activation was measured using the same method to the gold wire measurement.

For the measurement of gamma ray dose, thermo-luminescent dosimeters (TLDs) were used. Commercially available BeO powder TLD is usually encapsulated in borosilicate glass, which has a high sensitivity to thermal neutrons. Therefore, a special-ordered BeO TLD enclosed in a quartz glass capsule was used to measure the gamma ray dose rate in the phantom. This TLD has been used previously by Sakurai et. al at the Kyoto University Research Reactor [22] and was calibrated using a Co-60 source. Measurements were performed using a 10 cm, 12 cm, and a 15 cm diameter circular field.

### 2D water phantom simulation parameters

A computed tomography scan of a water phantom used for routine quality assurance (QA) (H: 28 cm, L: 21 cm, W: 21 cm with the phantom walls having a thickness of 1 cm (except for the front wall having a thickness of 2 mm) was performed. The image pixel size was 512 × 512 with a slice thickness of 2 mm. A uniform distribution of ^10^B with a concentration of 25 µg/g was set. The thermal neutron, epithermal neutron, fast neutron, and gamma ray distribution along the central beam axis was simulated for the three circular collimators (10, 12, and 15 cm diameter), which are available for clinical use.

1. NeuCure
  Calculations were performed with a voxel size of 3 mm^3^ (approximately 1 million voxels) and the Monte Carlo uncertainty was set to 5%.
2. SERA
  A voxel size of 1 cm^3^ with 4 × 10^6^ particles were simulated. The Monte Carlo uncertainty was not made available to the end user.
3. Independent PHITS model

A XYZ mesh size of 3 mm^3^ was set with approximately 1 × 10^10^ particles were simulated using simple sampling mode (i.e., event-generator mode was not used). The default parameters were used for the simulation and the thermal scattering law data S(α,β) library was utilised in describing the transport of thermal neutrons. The T-Track tally was used to obtain the neutron and gamma ray distribution inside the water phantom. The relative error, defined as the ratio of the standard deviation to the mean value of the tally result, was less than 0.5% for the thermal neutron flux, less than 5% for the fast neutron flux, and less than 1% for the gamma ray dose rate at a depth of 10 cm along the central beam axis. To compare the fast neutron component with experimental measurements, a small cylindrical region (diameter of 3 mm with a thickness of 0.1 mm) was modelled at the centre of the field, placed in front of the water phantom. The material of this geometry was set to indium with a density of 7.31 g/cm^3^. The T-Track with multiplier option was used to track the ^115^In(n,n’)^115m^In reaction inside the indium.

### 3D water phantom simulation comparison using gamma analysis

The 3D dose distribution of the boron dose, hydrogen dose and gamma ray dose component inside the water phantom calculated with the NeuCure was compared with the independent PHITS model calculation using 3D gamma analysis. An open source software, 3D slicer [23] was used to compare the 3D dose distribution. Details of the algorithm and the validation of the 3D slicer gamma analysis can be found elsewhere [24, 25]. The gamma calculation was performed with different dose difference/distance-to-agreement (DTA) criteria to examine the sources of discrepancies and their impact. Both extremities were tested, keeping the dose difference at a fixed value of 1% and varying the DTA, and keeping the DTA at a fixed value of 1 mm and varying the dose difference. A dose threshold of 10% was set for the calculation (Table 1).

**Table 1:** The mean dose inside the mock tumour and the brain and the maximum dose of the skin of an anthropomorphic phantom simulated using the three different systems for an irradiation time of 1 h assuming a uniform distribution of ^10^B concentration of 25 µg/g

Dose component	Mean dose (Gy)						Maximum dose (Gy)		
	Tumour			Brain			Skin		
	NeuCure	SERA	In-house	NeuCure	SERA	In-house	NeuCure	SERA	In-house
Boron	4.7	4.8	4.7	1.7	1.6	1.6	4.4	3.0	4.2
Gamma ray	2.3	2.5	2.4	1.4	1.3	1.2	2.1	2.1	2.1
Nitrogen	0.5	0.3	0.5	0.2	0.1	0.2	0.4	0.2	0.5
Hydrogen	0.3	0.3	0.3	0.1	0.2	0.1	1.6	1.7	1.8
Total biologically weighted dose (Gy_w_)	66.4	67.9	66.2	4.5	4.2	4.2	18.2	14.4	18.3

### Simulation of a brain BNCT using an anthropomorphic head phantom

An anthropomorphic head phantom (Therapy Body Phantom THRA-1, Kyoto Kagaku Co., Ltd.) was used to simulate a BNCT of the brain. A CT scan of the head phantom was performed (with the same scan parameters to the water phantom scan) and the images were imported into both RayStation and SERA TPS. The skull, brain, and skin region were contoured, and the corresponding material were set (shown in Table 2). The skin was defined as 3 mm inside from the external contour. The remaining region was set to tissue soft. An arbitrary 1 cm diameter spherical tumour was placed at a depth of 4 cm below the surface of the skin. A 12 cm diameter circular field entering the vertex of the phantom was set. The mean dose inside the mock tumour and the brain was calculated for an irradiation time of 1 h. The maximum dose delivered to the skin was also calculated. The dose calculation method is outlined in the appendix and the parameters used are summarised in Table 3. The same simulation parameters to the water phantom simulation were used.

**Table 2:** The elemental composition of each tissue type used for the simulation

Tissue type	Elemental composition (weight fraction)				
	H	C	N	O	Other
Soft tissue	0.101	0.111	0.026	0.762	–
Air	–	0.0001	0.755	0.232	0.013^a^
Bone	0.047	0.144	0.042	0.446	0.320^b^
Brain	0.107	0.145	0.022	0.712	0.014^c^
Skin	0.1	0.204	0.042	0.645	0.009^d^
Water	0.112	–	–	0.888	–

^a^Ar (0.013)

^b^Mg (0.002), P (0.105), S (0.003), Ca (0.210), Zn (0.0001)

^c^Na (0.002), P (0.004), S (0.002), Cl (0.003), K (0.003)

^d^Na(0.002), P(0.001), S(0.002), Cl(0.003), K(0.001)

**Table 3:** The CBE and RBE parameters used for the dose calculation

Tissue type	CBE	RBE_N_	RBE_H_	RBE_γ_	Tissue to blood ratio
Tumour	3.8 [34]	2.9	2.4	1	3.5 [35]
Skin	2.5 [36]	2.9	2.4	1	1
Bone	1	2.9	2.4	1	1
Brain	1.34 [37]	2.9	2.4	1	1
Soft tissue	1.34 [37]	2.9	2.4	1	1
Water	1	0	2.4	1	1
Air	0	0	0	0	0

For calculation using the independent PHITS model, the RT-PHITS (RadioTherapy package based on PHITS) functionality was used to convert the CT images of the anthropomorphic phantom into a voxel phantom. The same simulation parameters and beam configuration as above was used. The neutron and gamma ray flux inside each individual voxel was simulated and converted to dose using the corresponding KERMA coefficients. The simulated 3D dose distribution file was converted into a DICOM RT dose file and imported into the 3D slicer software for analysis. The RT dose file was overlayed onto the RT structure file (generated with RayStation) and the mean dose inside the tumour and brain and the maximum dose of the skin was determined.

## Results

**Fig. 3:**
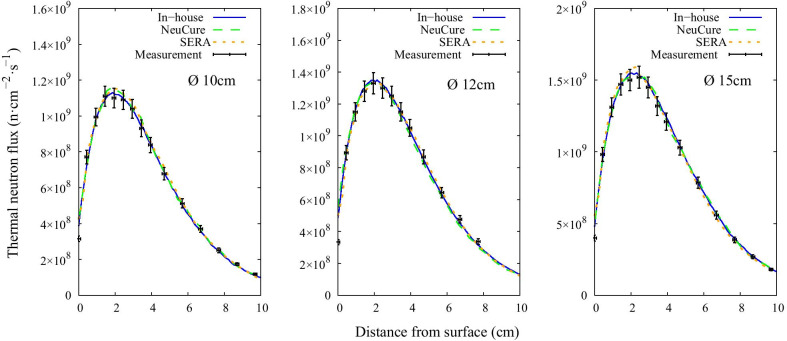
Thermal neutron flux along the central beam axis for each collimator size

**Fig. 4:**
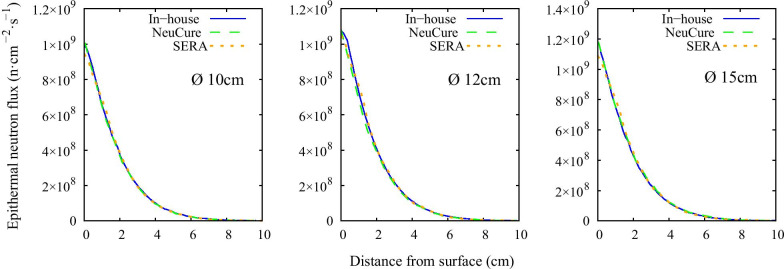
Epithermal neutron flux along the central beam axis for each collimator size

**Fig. 5:**
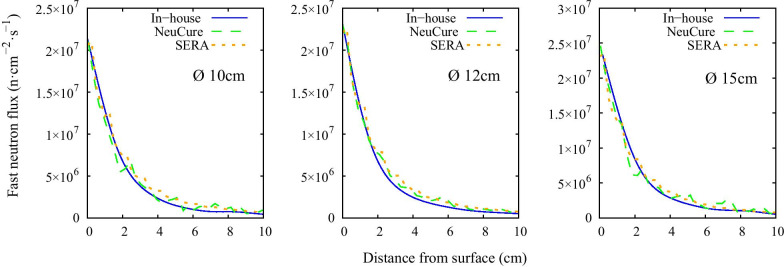
Fast neutron flux along the central beam axis for each collimator size

**Fig. 6:**
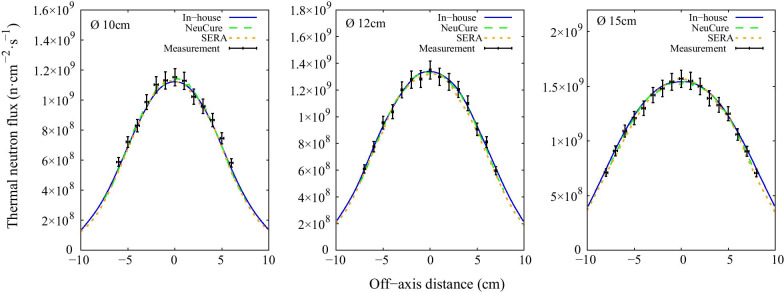
Off-axis thermal neutron flux at a depth of 2 cm inside the water phantom for each collimator size

**Fig. 7:**
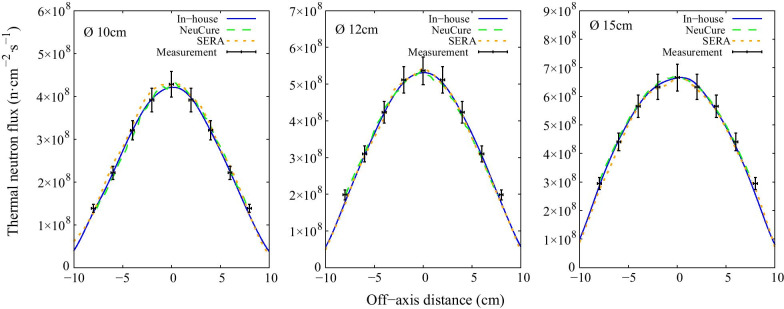
Off-axis thermal neutron flux at a depth of 6 cm inside the water phantom for each collimator size

**Fig. 8:**
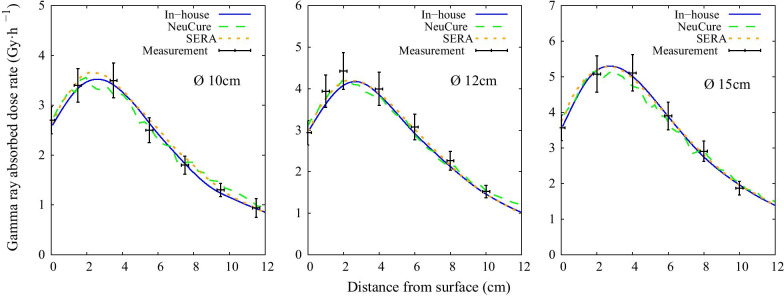
Gamma ray dose rate along the central beam axis for each collimator size

The thermal, epithermal, and fast neutron flux inside the water phantom along the central beam axis are shown in Fig. 3, Fig. 4, and Fig. 5, respectively. The off-axis thermal neutron flux inside the water phantom at a depth of 2 cm and 6 cm is shown in Fig. 6 and Fig. 7, respectively. The gamma ray dose rate inside the water phantom along the central beam axis is shown in Fig. 8. The experimentally measured values matched closely with the simulation results, within the corresponding experimental uncertainty (5% for gold foil activation and 10% for the TLDs). The (n,n’) reaction rate of the indium foil placed at the surface of the water phantom was measured to be (1.3 ± 0.22) × 10^–15^ per atom per coulomb and the PHITS simulation was calculated to be (1.5 ± 0.07) × 10^–15^ per atom per coulomb.

**Fig. 9:**
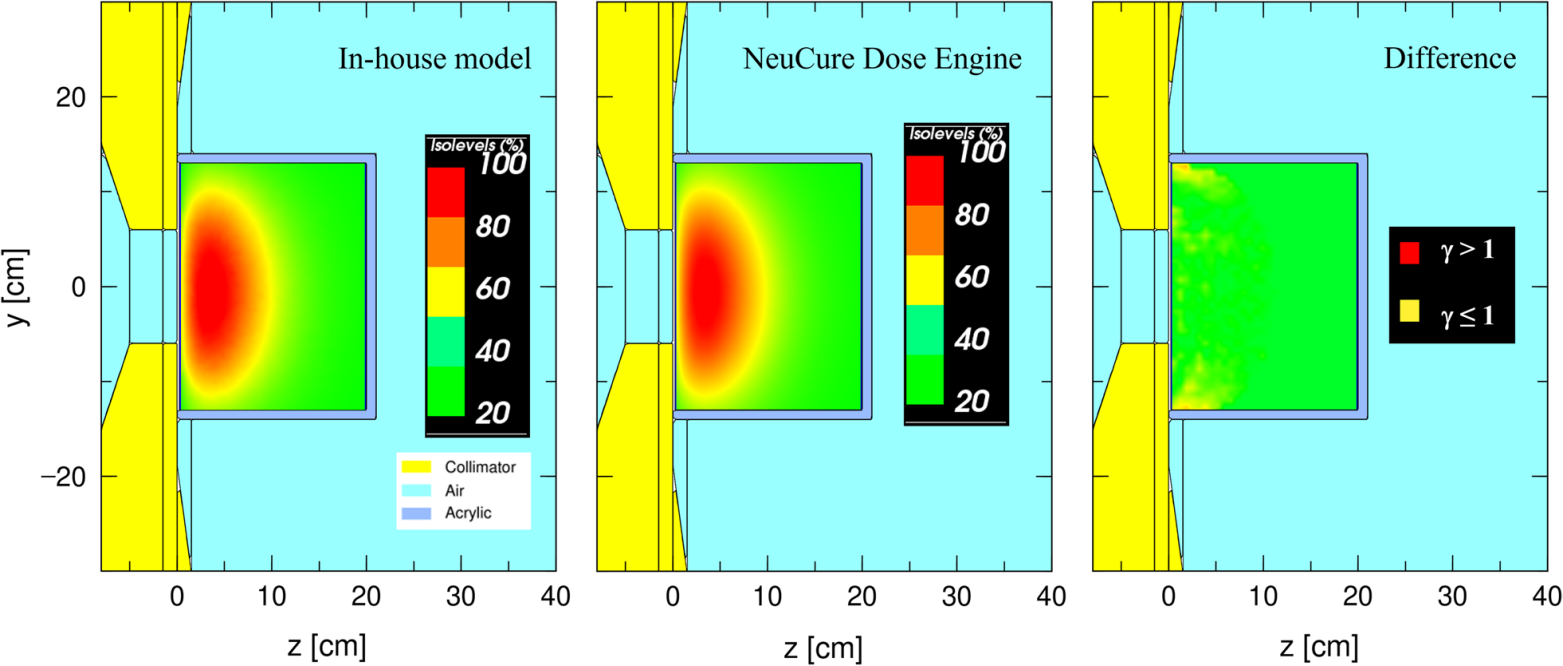
Boron dose distribution inside the water phantom for a 12 cm diameter collimator calculated with: left) in-house model. Middle) NeuCure and the difference at a 1 mm/2% criteria (Right)

**Fig. 10:**
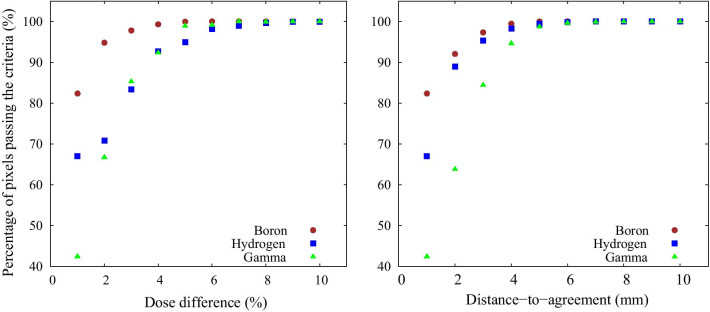
The number of pixels (%) passing the gamma analysis criteria for a fixed DTA of 1 mm (Left) and a fixed dose difference of 1% (right) for the three different dose components calculated with NeuCure and the in-house model

**Fig. 11:**
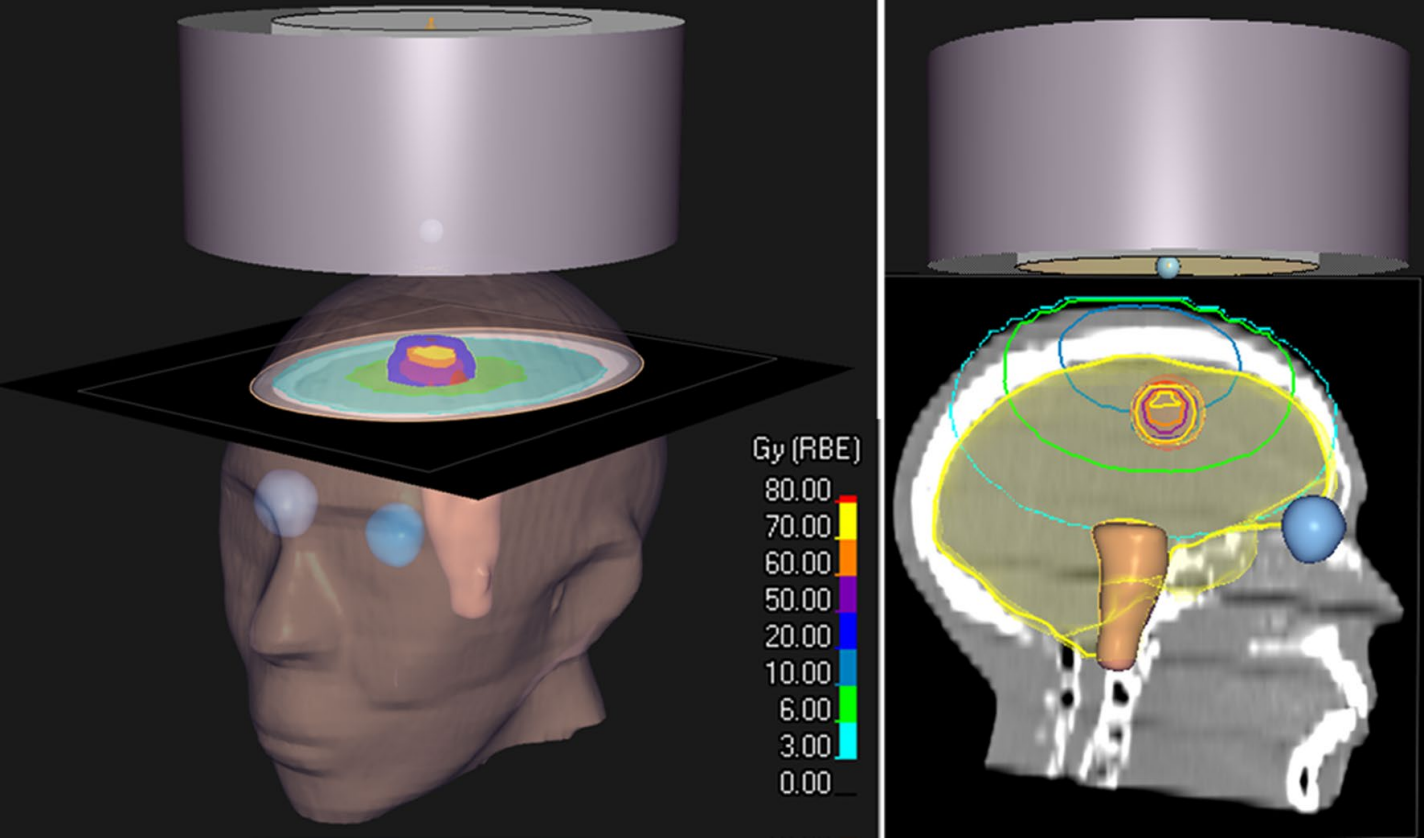
The reconstructed anthropomorphic phantom using RayStation, showing the beam entering the vertex of the phantom. The units are displayed as Gy(RBE) on RayStation, which is equivalent to Gy_w_ mentioned in this study

Figure 9 shows the boron dose distribution inside the water phantom simulated with the NeuCure and the in-house developed PHITS model. The gamma analysis results are shown in Fig. 10. The isodose distribution inside the head phantom calculated by NeuCure and displayed graphically by RayStation is shown in Fig. 11. Table 1 shows the mean dose inside the mock tumour and brain and the maximum dose of the skin of the anthropomorphic phantom calculated with the three different system.

## Discussion

To utilise the NeuCure for clinical use, no different to other TPS for external beam radiotherapy, a commissioning process is highly recommended to ensure accurate and precise dose delivery to the patient and to minimise the possibility of accidental exposure [26]. The system was compared against the BNCT TPS SERA, along with an in-house developed independent Monte Carlo dose calculation system and verification was performed with experimental measurements.

The thermal neutron flux and gamma ray dose rate inside the water phantom simulated with the different TPS and the in-house Monte Carlo system closely matched the experimental measurements. According to the JRC scientific and technical reports on the review and recommendations for the physical dosimetry of BNCT, the uncertainty of thermal neutron fluence determination by the gold wire activation method is approximately 5% [27]. Also, Kumada et al. performed a study to measure the thermal neutron flux inside a water phantom using a similar method as to the one performed in this study and reported an uncertainty of approximately 7%, with the major contributor being the efficiency calibration of the germanium detector (approximately 5%) [28]. Other sources of error include, but not limited to, self-absorption correction factor, phantom set-up and gold wire placement, and the mass measurement of the gold wire. The fast neutron component measured with the indium foil had a large experimental uncertainty (approximately 17%) primarily due to the low count rate. A longer irradiation time or increasing the thickness of the indium foil may increase the count rate, as a study performed by Pu et. al and Kobayashi et. al showed the self-shielding of gamma-rays by the indium foil was estimated to be negligible [29, 30].

The IAEA TecDoc 1223 summarised the total uncertainty to be approximately 5–7% for the determination of the thermal neutron flux, 15–20% for fast neutrons, and up to 20% for the gamma ray dose using TLDs in a mixed neutron-gamma irradiation field [21]. The sources of error for the gamma dose determination using TLD include, but not limited to, individual TLD characteristics, energy and angular dependence, TLD reader calibration, fading correction factor, thermal neutron sensitivity factor, self-absorption, phantom set-up and TLD placement error. The uncertainty may be reduced by precise calibration of each individual TLDs under a well-established irradiation condition.

The thermal neutron, epithermal neutron, and gamma ray flux along the central beam axis inside a water phantom simulated using the three different systems were found to be almost equivalent with one another (within 2%) up to a depth of 10 cm. The fast neutron flux, due to the relative low flux, the simulation uncertainty was high at a depth greater than a few centimetres.

The 3D gamma analysis of each dose component inside the water phantom was evaluated and a high pass rate (> 95%) for all components was achieved when the criteria was either 1 mm/5% or 5 mm/1%. The boron dose was found to have the highest pass rate (> 90%) for a tight criteria of 1 mm/2% and 2 mm/1%. The boron and gamma ray component showed no significant difference in the pass rate between the dose difference and DTA variation. On the contrary, the hydrogen component was found to have a large dependency on the DTA. This was because the hydrogen component is dependent on the fast neutron flux, which has a steep fall off as it enters the medium (water). Currently, there is no standard or recommendation for gamma analysis criteria for BNCT. Referring to the American Association of Physicists in Medicine (AAPM) report number 218, it is recommended that the gamma passing rate should be greater than 90%, with a 3%/2 mm criteria for IMRT QA verification [31]. Using the same criteria, the gamma pass rate for the boron, hydrogen and gamma dose was found to be 99.3%, 93.6%, and 91%, respectively. Majority of the failed points were found to be near the edge of the field, where the Monte Carlo uncertainty is relatively high compared to the centre of the field. As illustrated in the appendix, the largest contributor to the total BNCT dose is the boron dose component. Therefore, importance should be made on the accuracy of the boron dose component. The dose threshold was set to 10%, as recommended by AAPM. However, for BNCT, the total biologically weighted dose of the tumour is far greater than the surrounding normal tissue dose. When comparing the difference in the total dose using a dose threshold of 10%, a large portion of pixels outside the tumour region will be ignored. Therefore, it may be more worthwhile to perform the comparison separately (or compare only the total physical dose) to reduce the risk of excluding dose comparison at the normal tissue region.

The mean biologically weighted dose of the mock tumour and the brain of an anthropomorphic phantom was calculated using the three different systems. The percentage difference in the total mean biologically weighted dose of the tumour was found to be within 3%. The mean dose of the brain was also found to be similar between the three systems (a maximum deviation of 0.3 Gy_w_). For the maximum skin dose, a difference of less than 1% was found between NeuCure and the independent PHITS calculation. However, a large discrepancy was found with SERA (approximately a difference of 20% to the other systems). This large discrepancy could be due to the large voxel size (1 cm^3^) that SERA utilises and since the skin region is abutting the outer region, there may be some uncertainty in the interpolation between the voxels containing both outer region and skin material, which has been reported by Nigg [32]. There is also uncertainty in the region of interest (ROI) contoured with SERA and RayStation. Since the structures generated in SERA are of a different format to DICOM, the ROI contoured with SERA was not identical to the ROI generated by RayStation.

Currently, there is no method available to directly measure the dose of each component of a BNCT irradiation field. Therefore, performing a measurement to verify the treatment plan generated from a TPS as part of a patient specific routine QA cannot be performed at this stage. Therefore, a comparison to other dose calculation systems is the only check one can perform to gain confidence in the calculation performed by the TPS. According to the AAPM task group 114 [33], an independent dose calculation system, such as a secondary TPS, is recommended for non-IMRT clinical radiotherapy treatment plans. The in-house developed independent PHITS model has shown high agreement with the NeuCure system and may be used as an independent dose calculation check for patient plans generated with NeuCure. Also, as NeuCure is a commercialised product, the end user cannot change the code to suit their needs. Furthermore, to speed up the calculation time, parallel computing across multiple CPUs is currently not available with the commercial product. With the in-house system, configurations can be easily made, and multiple CPUs can be arranged to significantly reduce the calculation time. The in-house developed Monte Carlo calculation system will vastly increase the possibility of expanding the fundamental clinical research in BNCT.

## Conclusion

The NeuCure was compared with the existing BNCT treatment planning system, SERA, and with the in-house developed independent dose calculation system. A good agreement was found between the planning systems and the simulation results matched closely with the experimentally measured values. The in-house developed model may be used as a secondary validation tool for a patient treatment plan, verify when an update is performed on the NeuCure as part of an on-going quality assurance system, or to expand the fundamental clinical research in BNCT.

## Figures and Tables

**Fig. 12:**
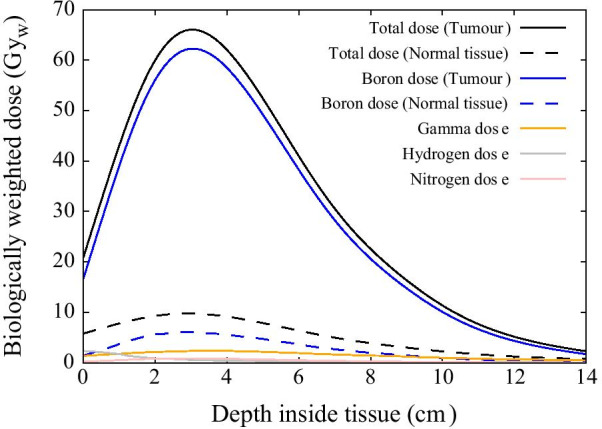
Central axis depth dose distribution inside a phantom assuming a uniform ^10^B concentration of 25 µg/g simulated using PHITS showing the different dose components present in the BNCT irradiation field for a dose limit of 10 Gy_w_ to the normal tissue

**Fig. 13:**
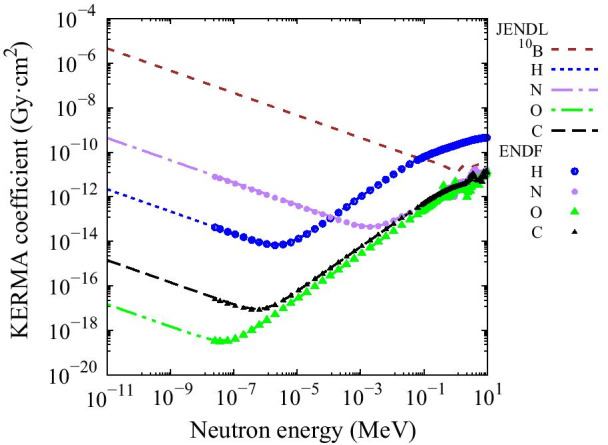
Neutron KERMA coefficient calculated by JENDL 4.0 and ENDF/B VI of the main elements of BNCT dose calculation

## Data Availability

The datasets used and/or analysed during the current study are available from the corresponding author on reasonable request.

## Appendix

In this paper, the dose of each component was calculated using the expression below,

$${D}_{x}=\int \varphi \left(E\right){K}_{x}\left(E\right) \, dE$$

where φ(E) is the fluence (cm^−2^), K(E) is the KERMA factor, and the subscript x denotes the element. The total dose delivered to a patient receiving BNCT is calculated by multiplying the absorbed dose of each individual radiation component present in the field with the corresponding Relative Biological Effectiveness (RBE) and taking the sum.

*D*_*T*_ = *CBE* × *D*_*B*_ + *RBE*_*H*_ × *D*_*H*_ + *RBE*_*N*_ × *D*_*N*_ + *RBE*_*γ*_ × *D*_*γ*_where D_T_ represents the total absorbed dose from each component, D_B_ is dose due to the ^10^B fission reaction (^10^B (n,α)^7^Li), D_H_ is the dose due to the epithermal and fast neutrons causing reoil protons from hydrogen in tissue, D_N_ is the dose due to the ^14^ N in tissue capturing a thermal neutron and emitting a proton in a ^14^C(n,α)^14^ N reaction, and D_γ_ is dose due to the gamma rays accompanying the neutron beam as well as gamma rays induced in the tissue itself. RBE_H_, RBE_N_ and RBE_γ_ are RBE values of hydrogen, nitrogen, and gamma rays, respectively. Compound Biological Effectiveness (CBE) considers the boron distribution inside a cell and is dependent on the boron compound type and the type of tissue being evaluated. The central axis depth dose distribution inside a cubic phantom (with the material set to ICRU soft tissue) for a 12 cm diameter collimator is shown in Fig. 11. The factors used to calculate the tumour and normal tissue doses are shown in Table 3 (Figs. 12, 13).

## Abbreviations

BNCT: Boron Neutron Capture Therapy
SERA: Simulation Environment for Radiation Applications
NCTPLAN: Neutron Capture Therapy Plan
PHITS: Particle and Heavy Ion Transport code System
MCNP: Monte Carlo N-Particle Transport
KERMA: Kinetic Energy Released in Matter
DICOM: Digital Imaging and Communications in Medicine
QA: Quality Assurance
TPS: Treatment Planning System
BSA: Beam Shaping Assembly
PMMA: Polymethyl methacrylate
TLD: Thermoluminescent dosimeter
CT: Computed Tomography
RBE: Relative Biological Effectiveness
CBE: Compound Biological Effectiveness
DTA: Distance to agreement
AAPM: American Association of Physicists in Medicine
